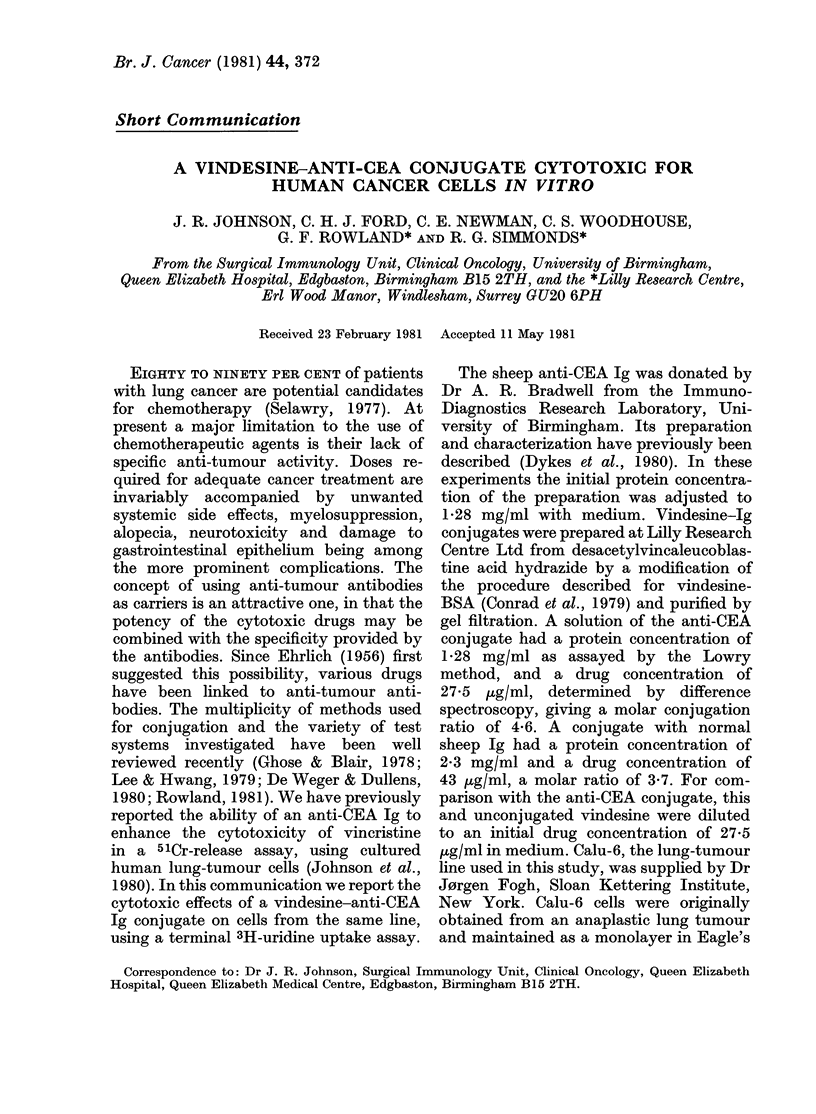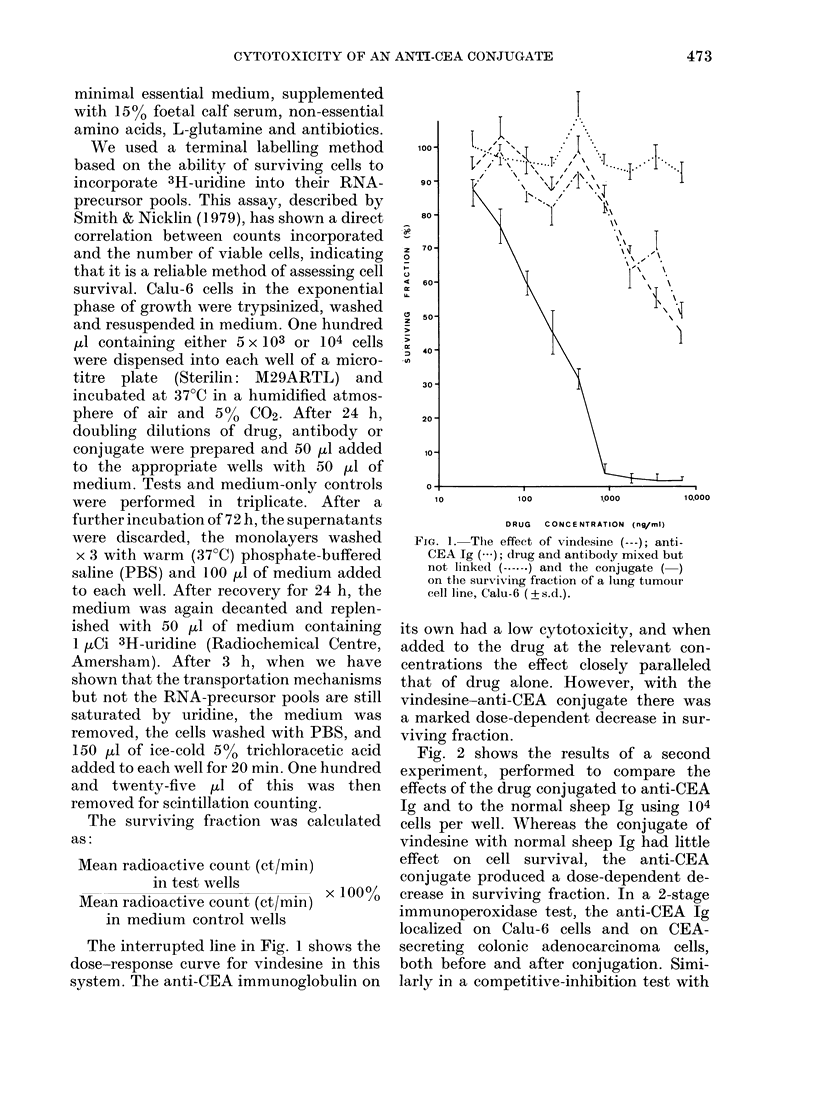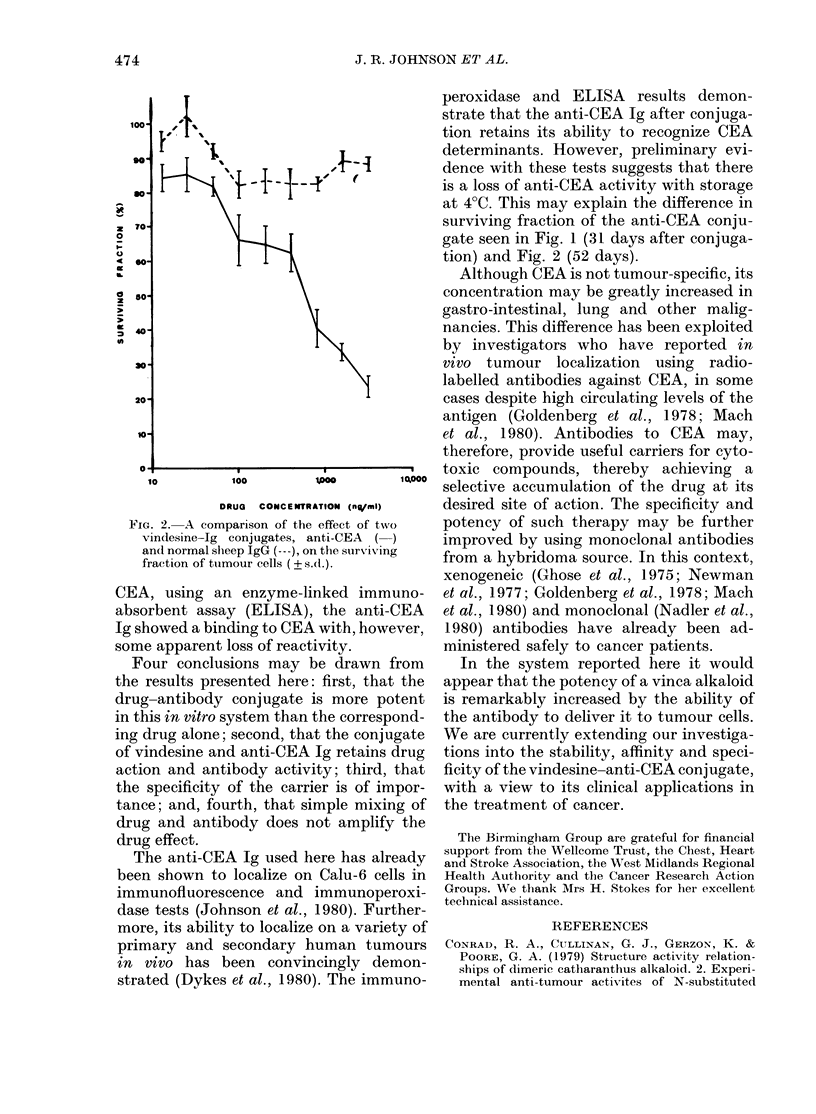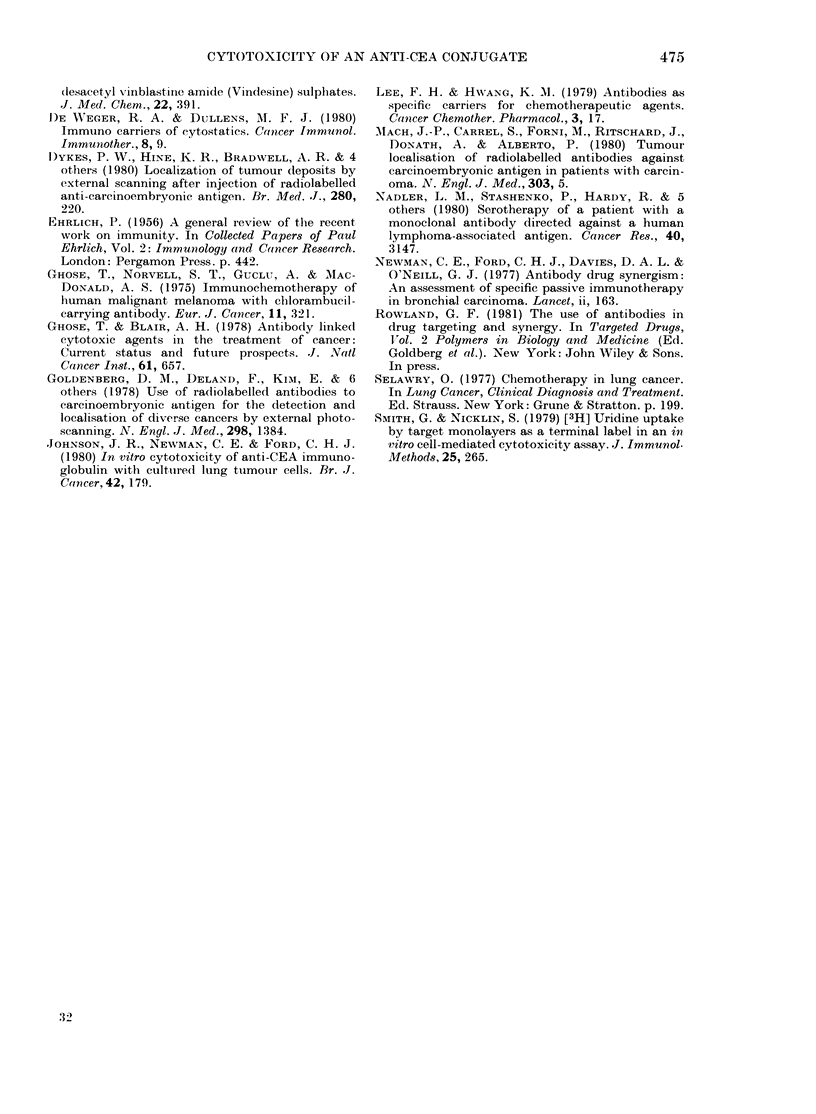# A vindesine-anti-CEA conjugate cytotoxic for human cancer cells in vitro.

**DOI:** 10.1038/bjc.1981.209

**Published:** 1981-09

**Authors:** J. R. Johnson, C. H. Ford, C. E. Newman, C. S. Woodhouse, G. F. Rowland, R. G. Simmonds


Br. J. Cancer (1981) 44, 372

### Short Communication

### A VINDESINE–ANTI-CEA CONJUGATE CYTOTOXIC FOR HUMAN CANCER CELLS *IN VITRO*

J. R. JOHNSON, C. H. J. FORD, C. E. NEWMAN, C. S. WOODHOUSE, G. F. ROWLAND* AND R. G. SIMMONDS*

*From the Surgical Immunology Unit, Clinical Oncology, University of Birmingham, Queen Elizabeth Hospital, Edgbaston, Birmingham B15 2TH, and the *Lilly Research Centre, Erl Wood Manor, Windlesham, Surrey GU20 6PH*

Received 23 February 1981 Accepted 11 May 1981

EIGHTY TO NINETY PER CENT of patients with lung cancer are potential candidates for chemotherapy (Selawry, 1977). At present a major limitation to the use of chemotherapeutic agents is their lack of specific anti-tumour activity. Doses required for adequate cancer treatment are invariably accompanied by unwanted systemic side effects, myelosuppression, alopecia, neurotoxicity and damage to gastrointestinal epithelium being among the more prominent complications. The concept of using anti-tumour antibodies as carriers is an attractive one, in that the potency of the cytotoxic drugs may be combined with the specificity provided by the antibodies. Since Ehrlich (1956) first suggested this possibility, various drugs have been linked to anti-tumour antibodies. The multiplicity of methods used for conjugation and the variety of test systems investigated have been well reviewed recently (Ghose & Blair, 1978; Lee & Hwang, 1979; De Weger & Dullens, 1980; Rowland, 1981). We have previously reported the ability of an anti-CEA Ig to enhance the cytotoxicity of vincristine in a $^{51}$Cr-release assay, using cultured human lung-tumour cells (Johnson *et al.*, 1980). In this communication we report the cytotoxic effects of a vindesine–anti-CEA Ig conjugate on cells from the same line, using a terminal $^{3}$H-uridine uptake assay.

The sheep anti-CEA Ig was donated by Dr A. R. Bradwell from the Immuno-Diagnostics Research Laboratory, University of Birmingham. Its preparation and characterization have previously been described (Dykes *et al.*, 1980). In these experiments the initial protein concentration of the preparation was adjusted to 1·28 mg/ml with medium. Vindesine–Ig conjugates were prepared at Lilly Research Centre Ltd from desacetylvincaleucoblastine acid hydrazide by a modification of the procedure described for vindesine-BSA (Conrad *et al.*, 1979) and purified by gel filtration. A solution of the anti-CEA conjugate had a protein concentration of 1·28 mg/ml as assayed by the Lowry method, and a drug concentration of 27·5 μg/ml, determined by difference spectroscopy, giving a molar conjugation ratio of 4·6. A conjugate with normal sheep Ig had a protein concentration of 2·3 mg/ml and a drug concentration of 43 μg/ml, a molar ratio of 3·7. For comparison with the anti-CEA conjugate, this and unconjugated vindesine were diluted to an initial drug concentration of 27·5 μg/ml in medium. Calu-6, the lung-tumour line used in this study, was supplied by Dr Jørgen Fogh, Sloan Kettering Institute, New York. Calu-6 cells were originally obtained from an anaplastic lung tumour and maintained as a monolayer in Eagle's

Correspondence to: Dr J. R. Johnson, Surgical Immunology Unit, Clinical Oncology, Queen Elizabeth Hospital, Queen Elizabeth Medical Centre, Edgbaston, Birmingham B15 2TH.

minimal essential medium, supplemented with 15% foetal calf serum, non-essential amino acids, L-glutamine and antibiotics.

We used a terminal labelling method based on the ability of surviving cells to incorporate $^3$H-uridine into their RNA-precursor pools. This assay, described by Smith & Nicklin (1979), has shown a direct correlation between counts incorporated and the number of viable cells, indicating that it is a reliable method of assessing cell survival. Calu-6 cells in the exponential phase of growth were trypsinized, washed and resuspended in medium. One hundred $\mu$l containing either $5 \times 10^3$ or $10^4$ cells were dispensed into each well of a microtitre plate (Sterilin: M29ARTL) and incubated at 37°C in a humidified atmosphere of air and 5% $CO_2$. After 24 h, doubling dilutions of drug, antibody or conjugate were prepared and 50 $\mu$l added to the appropriate wells with 50 $\mu$l of medium. Tests and medium-only controls were performed in triplicate. After a further incubation of 72 h, the supernatants were discarded, the monolayers washed $\times 3$ with warm (37°C) phosphate-buffered saline (PBS) and 100 $\mu$l of medium added to each well. After recovery for 24 h, the medium was again decanted and replenished with 50 $\mu$l of medium containing 1 $\mu$Ci $^3$H-uridine (Radiochemical Centre, Amersham). After 3 h, when we have shown that the transportation mechanisms but not the RNA-precursor pools are still saturated by uridine, the medium was removed, the cells washed with PBS, and 150 $\mu$l of ice-cold 5% trichloracetic acid added to each well for 20 min. One hundred and twenty-five $\mu$l of this was then removed for scintillation counting.

The surviving fraction was calculated as:

$$\frac{\text{Mean radioactive count (ct/min) in test wells}}{\text{Mean radioactive count (ct/min) in medium control wells}} \times 100\%$$

The interrupted line in Fig. 1 shows the dose–response curve for vindesine in this system. The anti-CEA immunoglobulin on its own had a low cytotoxicity, and when added to the drug at the relevant concentrations the effect closely paralleled that of drug alone. However, with the vindesine–anti-CEA conjugate there was a marked dose-dependent decrease in surviving fraction.

FIG. 1.—The effect of vindesine (---); anti-CEA Ig (···); drug and antibody mixed but not linked (-·-·-·) and the conjugate (—) on the surviving fraction of a lung tumour cell line, Calu-6 (±s.d.).

Fig. 2 shows the results of a second experiment, performed to compare the effects of the drug conjugated to anti-CEA Ig and to the normal sheep Ig using $10^4$ cells per well. Whereas the conjugate of vindesine with normal sheep Ig had little effect on cell survival, the anti-CEA conjugate produced a dose-dependent decrease in surviving fraction. In a 2-stage immunoperoxidase test, the anti-CEA Ig localized on Calu-6 cells and on CEA-secreting colonic adenocarcinoma cells, both before and after conjugation. Similarly in a competitive-inhibition test with

FIG. 2.—A comparison of the effect of two vindesine–Ig conjugates, anti-CEA (—) and normal sheep IgG (---), on the surviving fraction of tumour cells (±s.d.).

CEA, using an enzyme-linked immunoabsorbent assay (ELISA), the anti-CEA Ig showed a binding to CEA with, however, some apparent loss of reactivity.

Four conclusions may be drawn from the results presented here: first, that the drug–antibody conjugate is more potent in this *in vitro* system than the corresponding drug alone; second, that the conjugate of vindesine and anti-CEA Ig retains drug action and antibody activity; third, that the specificity of the carrier is of importance; and, fourth, that simple mixing of drug and antibody does not amplify the drug effect.

The anti-CEA Ig used here has already been shown to localize on Calu-6 cells in immunofluorescence and immunoperoxidase tests (Johnson *et al.*, 1980). Furthermore, its ability to localize on a variety of primary and secondary human tumours *in vivo* has been convincingly demonstrated (Dykes *et al.*, 1980). The immunoperoxidase and ELISA results demonstrate that the anti-CEA Ig after conjugation retains its ability to recognize CEA determinants. However, preliminary evidence with these tests suggests that there is a loss of anti-CEA activity with storage at 4°C. This may explain the difference in surviving fraction of the anti-CEA conjugate seen in Fig. 1 (31 days after conjugation) and Fig. 2 (52 days).

Although CEA is not tumour-specific, its concentration may be greatly increased in gastro-intestinal, lung and other malignancies. This difference has been exploited by investigators who have reported *in vivo* tumour localization using radiolabelled antibodies against CEA, in some cases despite high circulating levels of the antigen (Goldenberg *et al.*, 1978; Mach *et al.*, 1980). Antibodies to CEA may, therefore, provide useful carriers for cytotoxic compounds, thereby achieving a selective accumulation of the drug at its desired site of action. The specificity and potency of such therapy may be further improved by using monoclonal antibodies from a hybridoma source. In this context, xenogeneic (Ghose *et al.*, 1975; Newman *et al.*, 1977; Goldenberg *et al.*, 1978; Mach *et al.*, 1980) and monoclonal (Nadler *et al.*, 1980) antibodies have already been administered safely to cancer patients.

In the system reported here it would appear that the potency of a vinca alkaloid is remarkably increased by the ability of the antibody to deliver it to tumour cells. We are currently extending our investigations into the stability, affinity and specificity of the vindesine–anti-CEA conjugate, with a view to its clinical applications in the treatment of cancer.

The Birmingham Group are grateful for financial support from the Wellcome Trust, the Chest, Heart and Stroke Association, the West Midlands Regional Health Authority and the Cancer Research Action Groups. We thank Mrs H. Stokes for her excellent technical assistance.

### REFERENCES

CONRAD, R. A., CULLINAN, G. J., GERZON, K. & POORE, G. A. (1979) Structure activity relationships of dimeric catharanthus alkaloid. 2. Experimental anti-tumour activites of N-substituted desacetyl vinblastine amide (Vindesine) sulphates. *J. Med. Chem.*, **22**, 391.

DE WEGER, R. A. & DULLENS, M. F. J. (1980) Immuno carriers of cytostatics. *Cancer Immunol. Immunother.*, **8**, 9.

DYKES, P. W., HINE, K. R., BRADWELL, A. R. & 4 others (1980) Localization of tumour deposits by external scanning after injection of radiolabelled anti-carcinoembryonic antigen. *Br. Med. J.*, **280**, 220.

EHRLICH, P. (1956) A general review of the recent work on immunity. In *Collected Papers of Paul Ehrlich*, Vol. 2: *Immunology and Cancer Research.* London: Pergamon Press. p. 442.

GHOSE, T., NORVELL, S. T., GUCLU, A. & MACDONALD, A. S. (1975) Immunochemotherapy of human malignant melanoma with chlorambucil-carrying antibody. *Eur. J. Cancer*, **11**, 321.

GHOSE, T. & BLAIR, A. H. (1978) Antibody linked cytotoxic agents in the treatment of cancer: Current status and future prospects. *J. Natl Cancer Inst.*, **61**, 657.

GOLDENBERG, D. M., DELAND, F., KIM, E. & 6 others (1978) Use of radiolabelled antibodies to carcinoembryonic antigen for the detection and localisation of diverse cancers by external photoscanning. *N. Engl. J. Med.*, **298**, 1384.

JOHNSON, J. R., NEWMAN, C. E. & FORD, C. H. J. (1980) *In vitro* cytotoxicity of anti-CEA immunoglobulin with cultured lung tumour cells. *Br. J. Cancer*, **42**, 179.

LEE, F. H. & HWANG, K. M. (1979) Antibodies as specific carriers for chemotherapeutic agents. *Cancer Chemother. Pharmacol.*, **3**, 17.

MACH, J.-P., CARREL, S., FORNI, M., RITSCHARD, J., DONATH, A. & ALBERTO, P. (1980) Tumour localisation of radiolabelled antibodies against carcinoembryonic antigen in patients with carcinoma. *N. Engl. J. Med.*, **303**, 5.

NADLER, L. M., STASHENKO, P., HARDY, R. & 5 others (1980) Serotherapy of a patient with a monoclonal antibody directed against a human lymphoma-associated antigen. *Cancer Res.*, **40**, 3147.

NEWMAN, C. E., FORD, C. H. J., DAVIES, D. A. L. & O'NEILL, G. J. (1977) Antibody drug synergism: An assessment of specific passive immunotherapy in bronchial carcinoma. *Lancet*, ii, 163.

ROWLAND, G. F. (1981) The use of antibodies in drug targeting and synergy. In *Targeted Drugs, Vol. 2 Polymers in Biology and Medicine* (Ed. Goldberg *et al.*). New York: John Wiley & Sons. In press.

SELAWRY, O. (1977) Chemotherapy in lung cancer. In *Lung Cancer, Clinical Diagnosis and Treatment.* Ed. Strauss. New York: Grune & Stratton. p. 199.

SMITH, G. & NICKLIN, S. (1979) [$^{3}$H] Uridine uptake by target monolayers as a terminal label in an *in vitro* cell-mediated cytotoxicity assay. *J. Immunol. Methods*, **25**, 265.